# Supplementary material for: Genome-Wide Association Study of Milk Composition in Karachai Goats
Source: Animals (Basel). 2024 Jan 21;14(2):327. doi: 10.3390/ani14020327 (PMC10812594; doi:10.3390/ani14020327)
Supplement: Supplementary file 1 [file animals-14-00327-s001.zip › animals-2760852-supplementary.pdf]

animals MDPI

Table S1. Description of whole-genome and some suggestive* SNPs associated with fat content and fatty acids in the milk of Karachai goats

CHR	SNP	Position	P
TS			
2	snp18646-scaffold1882-539299	111 052 769	1.41E-06
3	snp11864-scaffold144-984620	46 725 075	9.00E-06
8	snp28090-scaffold300-3913146	39 751 214	9.65E-06
10	snp24669-scaffold251-1625913	56 089 166	6.55E-06
25	snp16908-scaffold1766-616140	1 861 790	7.79E-07
MSNF			
2	snp8325-scaffold130-2860751	6 443 953	1.47E-07
21	snp6514-scaffold1230-1489811	31 913 916	5.18E-06
PT			
2	snp8326-scaffold130-2909971	6394722	2.13E-07
2	snp8325-scaffold130-2860751	6443953	2.88E-06
8	snp997-scaffold1026-378556	70684382	1.34E-06
PC			
1	snp19495-scaffold197-240248	15 704 998	1.51E-06
2	snp8325-scaffold130-2860751	6 443 953	1.47E-07
8	snp43684-scaffold585-2375977	78 374 813	6.26E-06
8	snp43681-scaffold585-2255525	78 496 104	3.69E-07
8	snp997-scaffold1026-378556	70 684 382	9.99E-07
24	snp40879-scaffold526-1360443	44 527 159	7.88E-06
Cas. β			
2	snp8326-scaffold130-2909971	6 394 722	4.54E-08
2	snp8325-scaffold130-2860751	6 443 953	8.00E-07
Fat			
8	snp33285-scaffold391-913110	6 144 247	4.04E-06
25	snp16908-scaffold1766-616140	1 861 790	3.35E-06
Saturated fatty acids (SFA)			
2	snp18646-scaffold1882-539299	111 052 769	1.41E-06
3	snp10469-scaffold1373-2167084	52 769 710	9.19E-06
8	snp33285-scaffold391-913110	6 144 247	9.68E-06
13	snp8522-scaffold1308-1729010*	75 367 491	2.21E-05
16	snp50562-scaffold727-524836*	37 286 538	1.11E-05
23	snp10273-scaffold1368-2701834*	6 904 785	9.35E-05
Monounsaturated fatty acids (MUFA)			
1	snp27412-scaffold292-177570*	141 051 692	9.19E-05
13	snp8522-scaffold1308-1729010*	75 367 491	2.21E-05
25	snp16908-scaffold1766-616140*	1 861 790	1.23E-05
Polyunsaturated fat (Polyunsaturated fatty acids - PUFA)			
8	snp47195-scaffold66-1841919*	47 780 184	2.68E-05
16	snp18361-scaffold186-251734*	68 374 935	7.96E-05

23	snp10273-scaffold1368-2701834*	6 904 785	9.35E-05
Long-chain fatty acid (LCFA)			
4	snp1935-scaffold1053-1528396*	2 746 300	1.96E-05
8	snp33285-scaffold391-913110	6 144 247	9.68E-06
10	snp1441-scaffold104-780285*	25 486 826	5.54E-05
16	snp3754-scaffold112-3973504	63 371 269	5.51E-06
25	snp16908-scaffold1766-616140	1 861 790	7.79E-07
Medium chain fatty acid (MCFA)			
2	snp18646-scaffold1882-539299	111 052 769	1.41E-06
3	snp10469-scaffold1373-2167084	52 769 710	9.19E-06
10	snp24669-scaffold251-1625913	56 089 166	6.55E-06
16	snp50562-scaffold727-524836*	37 286 538	1.11E-05
23	snp10273-scaffold1368-2701834*	6 904 785	9.35E-05
25	snp16908-scaffold1766-616140	1 861 790	7.79E-07
Short-chain fatty acid (SCFA)			
2	snp18646-scaffold1882-539299	111 052 769	1.41E-06
3	snp10469-scaffold1373-2167084	52 769 710	9.19E-06
8	snp33285-scaffold391-913110	6 144 247	9.68E-06
23	snp10273-scaffold1368-2701834*	6 904 785	9.35E-05
25	snp16908-scaffold1766-616140	1 861 790	7.79E-07
Myristic C14:0			
2	snp18646-scaffold1882-539299	111 052 769	1.41E-06
3	snp10469-scaffold1373-2167084	52 769 710	9.19E-06
8	snp28090-scaffold300-3913146	39 751 214	9.65E-06
16	snp50562-scaffold727-524836*	37 286 538	1.11E-05
23	snp10273-scaffold1368-2701834*	6 904 785	9.35E-05
Palmitic C16:0			
2	snp18646-scaffold1882-539299	111 052 769	1.41E-06
3	snp10469-scaffold1373-2167084	52 769 710	9.19E-06
23	snp10273-scaffold1368-2701834*	6 904 785	9.35E-05
25	snp16908-scaffold1766-616140	1 861 790	7.79E-07
Oleic C18:1			
4	snp1935-scaffold1053-1528396*	2 746 300	1.96E-05
8	snp33285-scaffold391-913110	6 144 247	9.68E-06
10	snp1441-scaffold104-780285*	25 486 826	5.54E-05
13	snp8522-scaffold1308-1729010*	75 367 491	2.21E-05
16	snp3754-scaffold112-3973504	63 371 269	5.51E-06
25	snp16908-scaffold1766-616140	1 861 790	7.79E-07
Trans-isomers fatty acid			
8	snp47195-scaffold66-1841919*	47 780 184	2.68E-05
25	snp16908-scaffold1766-616140	1 861 790	7.79E-07
Lactose			
1	snp19495-scaffold197-240248	15 704 998	1.51E-06
1	snp23385-scaffold234-683659	25 509 749	2.58E-06

6	snp30534-scaffold337-667566	24 057 337	2.49E-06
8	snp997-scaffold1026-378556	70 684 382	9.99E-07
8	snp43685-scaffold585-2415660	78 335 071	5.99E-07
8	snp43681-scaffold585-2255525	78 496 104	3.69E-07
Acetone			
3	snp22179-scaffold219-771742	8 177 011	5.47E-06
7	snp21549-scaffold210-2243912	3 581 422	1.35E-06
9	snp43765-scaffold588-1398750	14 534 276	4.51E-06
27	snp51892-scaffold762-2837282	3 674 006	2.12E-06
Beta hydroxybutyrate (BHB)			
2	snp2511-scaffold1071-129957	14 902 755	9.44E-07
Urea			
2	snp25937-scaffold2682-105434	614 121	1.84E-06
3	snp11864-scaffold144-984620	46 725 075	9.00E-06
Feezing point depression (FPD)			
1	snp19495-scaffold197-240248	15 704 998	1.51E-06
1	snp23385-scaffold234-683659	25 509 749	2.58E-06
Acidity			
1	snp19495-scaffold197-240248	15 704 998	1.51E-06
1	snp23385-scaffold234-683659	25 509 749	2.58E-06
8	snp43684-scaffold585-2375977	78 374 813	6.26E-06
8	snp43681-scaffold585-2255525	78 496 104	3.69E-07

Table S2. Annotation of the identified candidate genes for the component composition of milk of Karachai goats

CHR	SNP$^{\pm 200\ kb}$	№ SNP – serial number of SNP in Figure 1	Trait	Gene	Biological function
1	snp27412-scaffold292-177570 $^{141251692…140851692}$	1	MUFA	*MX2*$^{141193475..141232778}$	protective response to the virus
2	snp18646-scaffold1882-539299$^{111252769…110852769}$	3	TS, MCFA, SFA C14, C16, SCFA	*METTL8*$^{1111185392..111270066}$	development of skeletal muscle tissue, differentiation of fat cells
2	snp9402-scaffold1341-2132092$^{114722555…114322555}$	4	BHB, Acetone	*ATF2*$^{114518050..114597771}$	development of skeletal muscle tissue, differentiation of fat cells
2	snp8314-scaffold130-	5	TS	*LUZP1*$^{6185376..6286457}$	development of the interventricular septum, bending

	2382340[7122494…6722494]				of neural folds, development of arteries
2	snp8322-scaffold130-2738985[6766020…6566020]	6	TFA	*HTR1D*[6305115..6307862]	smooth muscle contraction, chemical synaptic transmission, regulation of locomotion, vasoconstriction, regulation of behavior
2	snp25937-scaffold2682-105434[814121…414121]	7	Urea, TS	*NECAP2*[486514..500217]	endocytosis
2	snp8314-scaffold130-2382340[7122494…6722494]	8	TS	*ID3*[6650387..6652141]	development of the central nervous system, development of the heart
				HMGCL[6836125..6855444]	lipid metabolism, mitochondrial organization, process of biosynthesis of ketone bodies
2	snp2511-scaffold1071-129957[15102755…14702755]	9	BHB	*HPCA*[14931533..14940116]	development of the inner ear, cellular response to calcium ions, regulation of the activity of voltage-gated calcium channels
				FNDC5[14907902..14915966]	response to muscle activity, positive regulation of brown fat cell differentiation
2	snp47697-scaffold670-1960487[9886517…9486517]	10	MSNF	*SLC9A* [9830831..9883473]	cellular sodium ion homeostasis, pH regulation, response to muscle distribution, cellular response to acidic pH, cellular response to adrenaline stimulus, regulation of heart rate strength via cardiac conduction
3	snp22179-scaffold219-771742[8377011…7977011]	11	Acetone	*NEU2*[8043799..8057992]	catabolic process of glycoproteins, catabolic process of gangliosides, catabolic process of cellular oligosaccharides
				GIGYF2[8226338..8358229]	feeding behavior, locomotor behavior of adults, postembryonic development, growth of a multicellular organism, metabolic process of cellular protein, balance of neuromuscular processes
				INPP5D[7877943..8015888]	immune system process, longevity
3	snp48374-scaffold687-	12	TS	*PGM1*[38942586..39008777]	glucose metabolic process

	$1302080^{38979227...38579227}$				
3	snp55013-scaffold841-$1661526^{105681913...105281913}$	13	pH	*ARHGEF2*$^{105303668..105353888}$	cell morphogenesis, nervous system development, innate immune response
				LMNA$^{105450088..105478581}$	development of ventricular cardiac muscle cells
4	snp1935-scaffold1053-$1528396^{2946300...2546300}$	14	PUFA, LCFA, C18:1	*INSIG1*$^{2747017..2757803}$	triglyceride metabolic process, cholesterol biosynthesis process, cholesterol metabolism process, cholesterol homeostasis, negative regulation of fat cell differentiation, negative regulation of fatty acid biosynthesis process, palate development, cranial suture morphogenesis
				EN2 $^{2659430..2666542}$	midbrain development, hindbrain development, neuronal development, embryonic brain development
				PAXIP1$^{2922811..2963751}$	development of adipose tissue, chorion development
4	snp38614-scaffold49-$1209913^{110877395...110477395}$	15	Acetone	*CDK6*$^{110413189..110678573}$	response to the virus
5	snp47348-scaffold666-$52899^{97220952...96820952}$	16	Acetone	*ETV6*$^{96570433..96857059}$	vitellogenesis, neurogenesis
5	snp12158-scaffold1450-$249468^{107408739...107008739}$	17	Acetone	*CACNA1C*$^{107328368..107719644}$	immune system development, heart development
8	snp10589-scaffold1376-$2594525^{73806016...73406016}$	18	PC	*DPYSL2*$^{73547764..73663711}$	brain development
				ADRA1A$^{73747397..73862840}$	negative regulation of heart rate involved in baroreceptor response to increased systemic blood pressure, adult cardiac development, multicellular aging, regulation of cardiac muscle, fractional cells, cell growth in cardiac muscle cell development

8	snp43637-scaffold585-317127$^{80644212…80244212}$	19	pH	*DAPK1*$^{80466314..80672849}$	protective response to tumor cells, innate immune response, cellular response to organic matter
8	snp43681-scaffold585-2255525$^{78696104…78296104}$	20	pH, Lactose, PC, β-каз	*AGTPBP1*$^{78600749..78791306}$	neuromuscular process
8	snp47195-scaffold66-1841919$^{47980184…47580184}$	21	TFA, PUFA	*CEMIP2*$^{47675207..47756816}$	metabolic process
8	snp997-scaffold1026-378556$^{70884382…70484382}$	22	PC, Lactose, Cas. β, pH	*NKX3-1* 70600953..70606837	development of the heart, development of the dorsal aorta, development of the pharyngeal system
				NKX2-6 70639383..70643794	development of the hypothalamus, development of the embryonic heart tube, development of the tongue, development of the guiding tract, development of the cardiac muscle cells of the atria, development of the cardiac muscle cells of the ventricles, development of the pharyngeal system
				STC1 70791218..70806076	regulation of cardiac muscle, positive regulation of import and sources, negative regulation of renal phosphate excretion
8	snp34748-scaffold412-871719$^{91227795…90827795}$	23	FAT	*BAAT*$^{90845022..90861305}$	bile acid conjugation, glycine metabolic process, fatty acid metabolic process, bile acid biosynthesis process
				PLPPR1$^{90695441..90835782}$	process of phospholipid metabolism, development of the nervous system
8	snp28090-scaffold300-3913146$^{39951214…39551214}$	24	TS, PUFA, C14	*SLC1A* 39770036..39845247	behavioral response insurance, brain development, memory, sexual behavior, neurogenesis, adult behavior, morphogenesis of blood vessels
10	snp1441-scaffold104-	25	LCFA, C18	*FUT8*$^{25310706..25629694}$	left gas exchange

	780285$^{25686826…25286826}$				
10	snp24669-scaffold251-1625913$^{56289166…55889166}$	26	TS, PUFA, MCFA	*TPM1*	morphogenesis of cardiac muscle tissue of the ventricles, contraction of cardiac muscle
				LACTB	regulation of lipid metabolism
13	snp5221-scaffold1180-236240$^{36057527…35657527}$	27	PC, β-каз	*ODAD2*$^{35971967..36143096}$	heart development, development of the ventricular system
13	snp8522-scaffold1308-1729010$^{75567491…75167491}$	28	MUFA, C18, SFA	*EYA2*$^{75025156..75283017}$	development of striated muscle tissue
16	snp18361-scaffold186-251734$^{68574935…68174935}$	29	PUFA	*PROX1*$^{68179003..68234777}$	determination of cell development/death, kidney development
16	snp50562-scaffold727-524836$^{37486538…37086538}$	30	TS, SFA, MCFA, C14	*FMO2*$^{37286455..37330116}$	acid determination metabolic process, toxin metabolic process, oxygen metabolic process
				FMO1$^{37336303..37376884}$	metabolic process of acid determination, metabolic process of xenobiotics, metabolic process of toxins
16	snp3754-scaffold112-3973504$^{63571269…63171269}$	31	LCFA, C18, MUFA	*NCF2*$^{63224955..63263716}$	phagocytosis, "oxygen explosion", trophy of neutral leukocytes in the pathogenesis of the inflammatory process
16	snp8683-scaffold131-4589642$^{54997494…54597494}$	32	FAT, PT	*TNN*$^{54831011..54903426}$	regulation of bone development
18	snp18289-scaffold1857-308667$^{56470790…56070790}$	33	Urea	*SULT2B1*$^{56239547..56275810}$	cholesterol metabolic process
				GRIN2D$^{56117047..56151568}$	reaction to fear, motor behavior of adults, regulation of sensory perception of pain
				SPHK2$^{56292056..56299987}$	blood vessel development, brain development
				NTN5$^{56321271..56326460}$	neurogenesis
				IZUMO1$^{56393899..56396881}$	fusion of sperm with the plasma membrane of the egg, sperm-egg recognition

				FUT1$^{56399123..56402123}$	metabolic process, protein glycosylation
21	snp6484-scaffold1230-240156$^{33359659...32959659}$	34	MSNF	*MPI*$^{33246409..33253730}$	metabolic process of carbohydrates
23	snp10273-scaffold1368-2701834$^{7104785...6704785}$	35	TS, C14, C16, MCFA, PUFA, SFA, SCFA	*PHACTR1*$^{6556030..7073500}$	development of the cerebral cortex
23	snp48737-scaffold692-158314$^{45730320...45330320}$	36	PC	*BAG2*$^{45658115..45668381}$	chemical protein process
25	snp16907-scaffold1766-582489$^{2027754...1627754}$ snp16908-scaffold1766-616140$^{2061790...1661790}$	37	TS, SFA, PUFA, MUFA, MCFA, C16, C18:1, LCFA, C14, SCFA, TFA	*ECI1*$^{1672290..1685556}$	beta-oxidation of fatty acids
				PGP$^{1649864..1652731}$	glycerol biosynthesis process
		38		*ABCA3*$^{1701870..1743075}$	positive regulation of cholesterol efflux, lung development, phosphatidylcholine metabolic process, phosphatidylglycerol metabolic process, lipid biosynthesis regulation process, phospholipid homeostasis
				AMDHD2$^{1915270..1921956}$	metabolic process of carbohydrates
				PDPK1$^{1932902..1996678}$	pancreatic type B cell development, cellular response to epidermal growth factor stimulus
26	snp18573-scaffold1878-337881$^{14293322...13893322}$	39	Cas. β	*PDZD8*$^{14290884..14368219}$	pancreatic type B cell development, cellular response to epidermal growth factor stimulus
26	snp41130-scaffold532-1727870$^{30209081...29809081}$	40	SCFA	*SEMA4G*$^{29853719..29867663}$	maintenance of the nervous system, cell differentiation
26	snp47577-scaffold67-3351554$^{17989628...17589628}$	41	Cas. β, PC	*CASP7*$^{17808414..17850585}$	heart development
				NRAP$^{32471325..32547298}$	tendon joint of the thigh
				HABP2$^{17935249..17970574}$	blood clotting

27	snp51881-scaffold762-2349319[4343759…3943759]	42	Acetone	*THRB*[3763978..4201001]	positive regulation of thyroid hormone-mediated signaling pathway, sensory sound perception, negative regulation of female receptivity
27	snp55772-scaffold864-4012988[20994598…20594598]	43	MSNF, Cas. β	*MFHAS1*[20648690..20757838]	reaction to an inflammatory process in the body

Note*: CHR – chromosome, SNP – single nucleotide polymorphism; genes located within SNPs are highlighted in bold.
Fat – fat content; PT – true protein content; PC – crude protein content; Lactose – lactose; MSNF – milk solids not fat ; TS – total solids; Cas. β – casein; BHB - hydroxy-butyrate; C14:0 – myristic FA; C16:0 – palmitic FA; C18:1 – oleic FA; LCFA – long chain fatty acids; MCFA – medium chain fatty acids; MCFA – monounsaturated fatty acids; PUFA – polyunsaturated fatty acids; SFA – saturated fatty acids; SCFA – short chain fatty acids; TFA – trans-isomers of fatty acids.
